# Magnesium Absorption in Intestinal Cells: Evidence of Cross-Talk between EGF and TRPM6 and Novel Implications for Cetuximab Therapy

**DOI:** 10.3390/nu12113277

**Published:** 2020-10-26

**Authors:** Giuseppe Pietropaolo, Daniela Pugliese, Alessandro Armuzzi, Luisa Guidi, Antonio Gasbarrini, Gian Lodovico Rapaccini, Federica I. Wolf, Valentina Trapani

**Affiliations:** 1 Sezione di Patologia Generale, Dipartimento di Medicina e Chirurgia Traslazionale, Fondazione Policlinico Universitario A. Gemelli IRCCS—Università Cattolica del Sacro Cuore, 00168 Rome, Italy; giuseppe.pietropaolo@uniroma1.it; 2 UOC Medicina Interna e Gastroenterologia, Dipartimento di Medicina e Chirurgia Traslazionale, Fondazione Policlinico Universitario A. Gemelli IRCCS—Università Cattolica del Sacro Cuore, 00168 Rome, Italy; daniela.pugliese@policlinicogemelli.it (D.P.); alessandro.armuzzi@unicatt.it (A.A.); luisa.guidi@unicatt.it (L.G.); antonio.gasbarrini@unicatt.it (A.G.); gianludovico.rapaccini@policlinicogemelli.it (G.L.R.)

**Keywords:** biomarker, colorectal cancer, EGFR, hypomagnesemia, magnesium supplementation, monoclonal antibodies, targeted therapy

## Abstract

Hypomagnesemia is very commonly observed in cancer patients, most frequently in association with therapy with cetuximab (CTX), a monoclonal antibody targeting the epithelial growth factor receptor (EGFR). CTX-induced hypomagnesemia has been ascribed to renal magnesium (Mg) wasting. Here, we sought to clarify whether CTX may also influence intestinal Mg absorption and if Mg supplementation may interfere with CTX activity. We used human colon carcinoma CaCo-2 cells as an in vitro model to study the mechanisms underlying Mg transport and CTX activity. Our findings demonstrate that TRPM6 is the key channel that mediates Mg influx in intestinal cells and that EGF stimulates such influx; consequently, CTX downregulates TRPM6-mediated Mg influx by interfering with EGF signaling. Moreover, we show that Mg supplementation does not modify either the CTX IC50 or CTX-dependent inhibition of ERK1/2 phosphorylation. Our results suggest that reduced Mg absorption in the intestine may contribute to the severe hypomagnesemia that occurs in CTX-treated patients, and Mg supplementation may represent a safe and effective nutritional intervention to restore Mg status without impairing the CTX efficacy.

## 1. Introduction

Nutritional deficits, defined as an imbalance between intake and metabolic requirements, are very common in cancer patients and may be caused by both the tumor itself and its treatment [1]. Appropriate nutritional interventions that correct such an imbalance reduce the risk of interruption or discontinuous treatment and improve quality of life [2]. Hypomagnesemia is frequent in oncologic patients, especially in those subjected to cisplatin-based therapies [3]. More recently, hypomagnesemia has emerged as the most notable adverse effect of the anti-EGFR monoclonal antibody cetuximab (CTX), which is widely used for advanced colorectal cancer (CRC) [4]. Several meta-analyses have shown that the incidence of all-grade hypomagnesemia in CTX-treated patients could be as high as about 35%; in about 5% of cases, hypomagnesemia can be severe (grade 3–4) and cause symptoms that require magnesium (Mg) supplementation [5,6,7,8]. On the other hand, early hypomagnesemia seems to act as a good predictor of the efficacy and outcome of CTX in *KRAS* wild-type CRC patients [9]. Although opposing results have also been reported [10], a recent meta-analysis confirmed that hypomagnesemia is associated with better progression-free survival, overall survival, and overall relative risk in CTX-treated *KRAS* wild-type CRC patients [11]. In addition to the clinical relevance of these findings, Vincenzi et al. [12] went as far as proposing that reduced serum Mg levels might potentiate the chemotherapeutic effects of CTX, which raised an intense debate among the scientific community [13,14]. 

Mg is a micronutrient involved in a plethora of cell functions, acting as a cofactor for a multitude of enzymes [15]. Systemic Mg homeostasis depends on the concomitant action of the intestine, responsible for Mg uptake from food, and the kidneys, which regulate Mg excretion. Magnesium is absorbed through different mechanisms, including passive paracellular transport, which is driven by the electrochemical gradient, and active transcellular transport, which is mediated by two highly homologous Mg channels—transient receptor potential melastatin (TRPM) channels type 6 and 7. TRPM7 is ubiquitously expressed, while TRPM6 is mainly expressed in the kidneys, the distal small intestine, and the colon [16]. Although the distal convoluted tubule of the kidney has long been considered the key gatekeeper of systemic Mg, the latest findings challenged such a view and suggested that intestinal Mg uptake might be of primary relevance [17]. Recent results corroborate the view that TRPM6, rather than TRPM7, modulates magnesium homeostasis in the colon. Ferioli et al. reported that TRPM6 function cannot be replaced by other channels [18]. Likewise, our group demonstrated that, in colon mucosa, TRPM6 is responsible for Mg influx and cell proliferation leading to mucosal healing [19,20].

The present view is that CTX-induced hypomagnesemia originates from molecular cross-talk between the EGF pathway and the regulatory mechanism for systemic Mg homeostasis in the kidneys. Such interaction was elucidated through the discovery of a mutation in the *EGF* gene in a rare genetic condition characterized by renal Mg wasting [21] and the following molecular characterization [22]. It was demonstrated that EGF acts as a magnesiotropic hormone by stimulating the surface expression and activity of the TRPM6 channel on the apical membrane of kidney epithelial cells, which in turn mediates Mg uptake. Therefore, by antagonizing EGF, ultimately CTX inhibits renal Mg reabsorption by TRPM6 and alters the whole-body Mg balance. 

In addition to its well-established role in tumor growth and progression, EGF is also an important actor in intestinal development and mucosal repair [23]. Furthermore, EGF has been shown to be an important regulator of the expression, trafficking, and activity of epithelial transport proteins in the intestine [24]. However, despite the current emphasis on the importance of gut absorption for Mg homeostasis, the molecular cross-talk between EGF and TRPM6 has never been investigated in intestinal epithelial cells so far, nor is it known whether CTX can also affect the intestinal Mg absorption. Moreover, evidence that hypomagnesemia may serve as a positive prognostic factor in CTX-treated patients strongly contrasts with the fact that hypomagnesemia may cause severe discomfort and may even pose a serious threat to their lives. In this context, a crucial issue remains unanswered regarding the possibility that Mg supplementation may interfere with CTX efficacy. In the present paper, we sought to clarify two pressing issues that might have important clinical implications for CTX therapy: (1) the role of altered intestinal Mg absorption in the development of CTX-induced hypomagnesaemia; (2) the effect of Mg supplementation on the efficacy of CTX treatment.

## 2. Materials and Methods 

### 2.1. Cell Culture

The constitutive activation of the MAPK pathway may limit the effectiveness of CTX treatment [25]. We screened different colon cancer cell lines and chose human colon carcinoma CaCo-2 cells, which harbor wild-type forms of critical genes, such as *KRAS*, *BRAF*, *PI3K3CA*, and *PTEN* [26]. CaCo-2 cells also express EGFR [27]. Cells were routinely grown in Dulbecco’s modified Eagle’s medium (DMEM) supplemented with 20% fetal bovine serum (FBS), 2mM of glutamine, 100 U/mL of penicillin, and 100 μg/mL of streptomycin in a 5% CO_2_ humidified atmosphere at 37 °C. The reagents for cell culture were from Euroclone (Pero, Milan, Italy). Recombinant EGF was purchased from PeproTech (London, UK) and used at a concentration of 10 ng/mL, as previously reported [28]. Before EGF stimulation, the cells were starved in FBS-free medium for 24 h. Cetuximab was kindly provided by the Oncology Pharmacy Unit, “Agostino Gemelli” University Hospital, and used at an optimal concentration of 70 μg/mL, as inferred from cytotoxicity assays (see Section 2.2 and Section 3.1). To obtain a transient downregulation of TRPM6, predesigned siRNA against human *TRPM6* was purchased from Qiagen. Specific siRNAs were transfected into cells (1300 ng per 400,000 cells) using HiPerFect Transfection Reagent (Qiagen, Milan, Italy) following the manufacturer’s protocol (https://www.qiagen.com/it/transfectionprotocols/transfectionprotocol/). Non-silencing scrambled sequences were used as controls.

### 2.2. MTT Cytotoxicity Assay

Cells were seeded in 24-well plates at a density of 40,000 cells/well and allowed to adhere for 24 h before drug treatment. CTX (concentration range: 7.5 μg/mL to 240 μg/mL) was added to the culture medium in triplicates. After 24 h, the culture medium was replaced with serum-free medium containing 3-[4,5-dimethylthiazol-2-yl]-2,5-diphenyltetrazolium bromide (MTT, 1 mg/mL), and cells were incubated for 90 min at 37 °C. Finally, formazan crystals were dissolved in acidified isopropanol (0.04 N HCl in isopropanol), and the absorbance was read at λ = 565 nm. Data were analyzed using Prism software (version 5.01, GraphPad Software Inc., La Jolla, CA, USA) and dose–response curves were obtained by nonlinear regression (sigmoidal curve, variable slope). 

### 2.3. Western Blotting

Cells were lysed in RIPA buffer (50 mM of Tris, pH 8, 150 mM of NaCl, 1 mM of EDTA, 1% NP-40, 0.05% sodium deoxycholate, 0.1% SDS) supplemented with protease and phosphatase inhibitors (Halt™ inhibitor cocktail, ThermoFisher Scientific, Milan, Italy). Protein concentrations were determined using the Bradford protein assay (Bio-Rad). Cell extracts were resolved by SDS-PAGE; transferred to PVDF membranes; and probed with rabbit polyclonal anti-TRPM6 (1:500, Biorbyt), anti-ERK1/2 (1:1000, Cell Signaling Technology), anti phospho-ERK1/2 (1:1000, Cell Signaling Technology), or anti-β-actin (1:1000, Sigma-Aldrich) primary antibodies. Horseradish peroxidase-conjugated secondary antibodies (GE Healthcare) were detected by the ECL Prime Western Blotting Detection Reagent (GE Healthcare) and the ChemiDoc XRS system (Bio-Rad). Densitometric analysis was performed by the ImageJ software (NIH, http://imagej.nih.gov/ij/).

### 2.4. Mg Influx Measurements

Subconfluent cells grown on 35 mm microscopy dishes (μ-dish, ibidi GmbH) were loaded with 3 μM of Mag-Fluo-4-AM (ThermoFisher Scientific), and imaged in a Na^+^, Ca^2+^, and Mg^2+^-free buffer at a confocal laser scanning microscope (Nikon A1 MP), as previously described [28]. Cytosolic fluorescence signals were recorded as time series at a sampling frequency of 30 frames/min. The baseline was monitored for 30 s, then MgSO_4_ was added drop-wise to a final concentration of 5 mM. Changes in the intracellular Mg levels at the single-cell level were estimated by the mean fluorescent increment ΔF/F [29]. Image analysis was performed by the NIS-Elements Confocal Software on 10 representative cells in each microscopic field, and experiments were repeated independently at least three times.

### 2.5. Statistical Analyses

All the experiments were repeated independently three times. The Prism software (version 5.01, GraphPad Software Inc., La Jolla, CA, USA) was used for all the statistical analyses. Statistical significance was evaluated using Student’s t-test, when comparing two groups; one-way ANOVA, when comparing more groups in relation to only one variable; and two-way ANOVA, when comparing more groups in relation to two variables. ANOVA was followed by Bonferroni’s test. Differences were considered statistically significant for *p*-values < 0.05, and significance levels were assigned as follows: * for *p* < 0.05, ** for *p* < 0.01.

## 3. Results

### 3.1. TRPM6 Mediates Mg Influx in CTX-Sensitive CaCo-2 Cells

**Figure 1:**
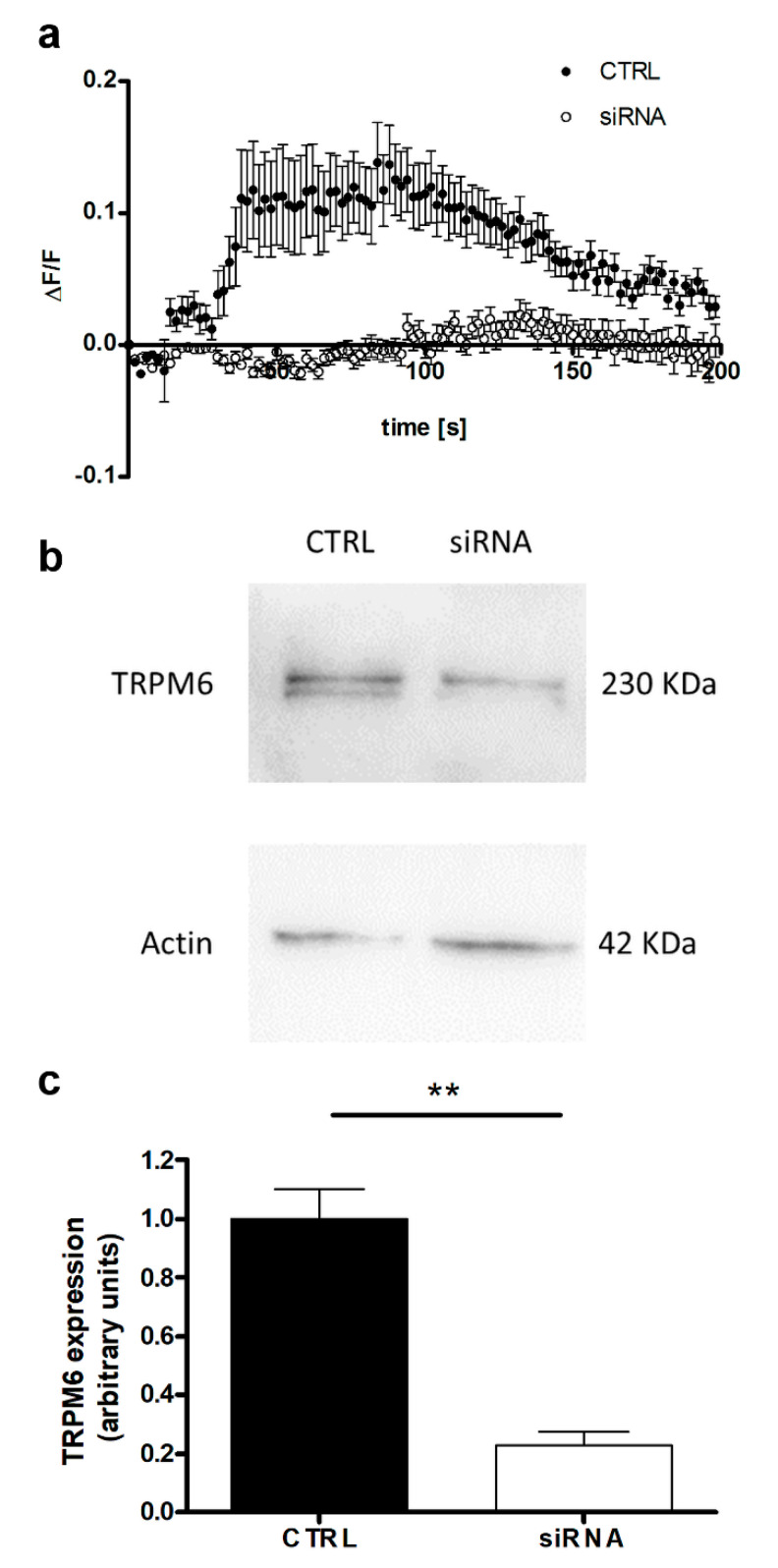
The TRPM6 channel mediates the Mg influx in human colon carcinoma cells. CaCo-2 cells were transiently silenced for TRPM6 (siRNA) and assessed 48h after transfection. Control cells (CTRL) received non-silencing scrambled siRNA. (**a**) Mg influx capacity in TRPM6-silenced (open circles) vs. control cells (solid circles), as assessed by the live imaging of Mag-Fluo-4-loaded cells; a representative experiment is shown. (**b**) TRPM6 protein expression in *TRPM6*-silenced and control cells, as evaluated by Western blot analysis; a representative blot is shown. (**c**) Quantification of TRPM6 protein expression by Western blot densitometry normalized to β-actin levels (*n* = 3, mean ± SD) in TRPM6-silenced (white bar) and control (black bar) cells. ** *p* < 0.01 by paired Student’s t-test. Full scans of original blots are available in Figure S1.

First, we assessed the sensitivity of CaCo-2 cells to CTX with an MTT assay. The IC50 at 24h was (66 ± 16) μg/mL; therefore, in the following experiments, a CTX dose of 70 μg/mL was used. Next, we characterized the basal Mg influx capacity by the live imaging of CaCo-2 cells loaded with the Mg-specific fluorescent probe Mag-Fluo-4. The addition of 5 mM of MgSO_4_ to the extracellular medium induced a rapid increase in fluorescence (i.e., intracellular Mg concentration) up to about 10% of the basal level; the fluorescence then gradually decreased to basal levels within about 3 min (Figure 1a, solid circles). CaCo-2 cells express the TRPM6 channel [19]. To determine whether the detected Mg influx is mediated by TRPM6, we repeated the same experiment in *TRPM6*-silenced CaCo-2 cells. Silencing by transient siRNA transfection significantly decreased the TRPM6 protein levels, as assessed by Western blot analysis at 48h (Figure 1b,c). After 48 h from siRNA transfection, the Mg influx upon the addition of extracellular MgSO_4_ was nearly abolished in *TRPM6*-silenced cells in comparison with the control cells (Figure 1a, open circles). These results prove that TRPM6 is the key channel that mediates the Mg influx in CaCo-2 cells.

### 3.2. Cetuximab and EGF Modulate Mg Influx

**Figure 2:**
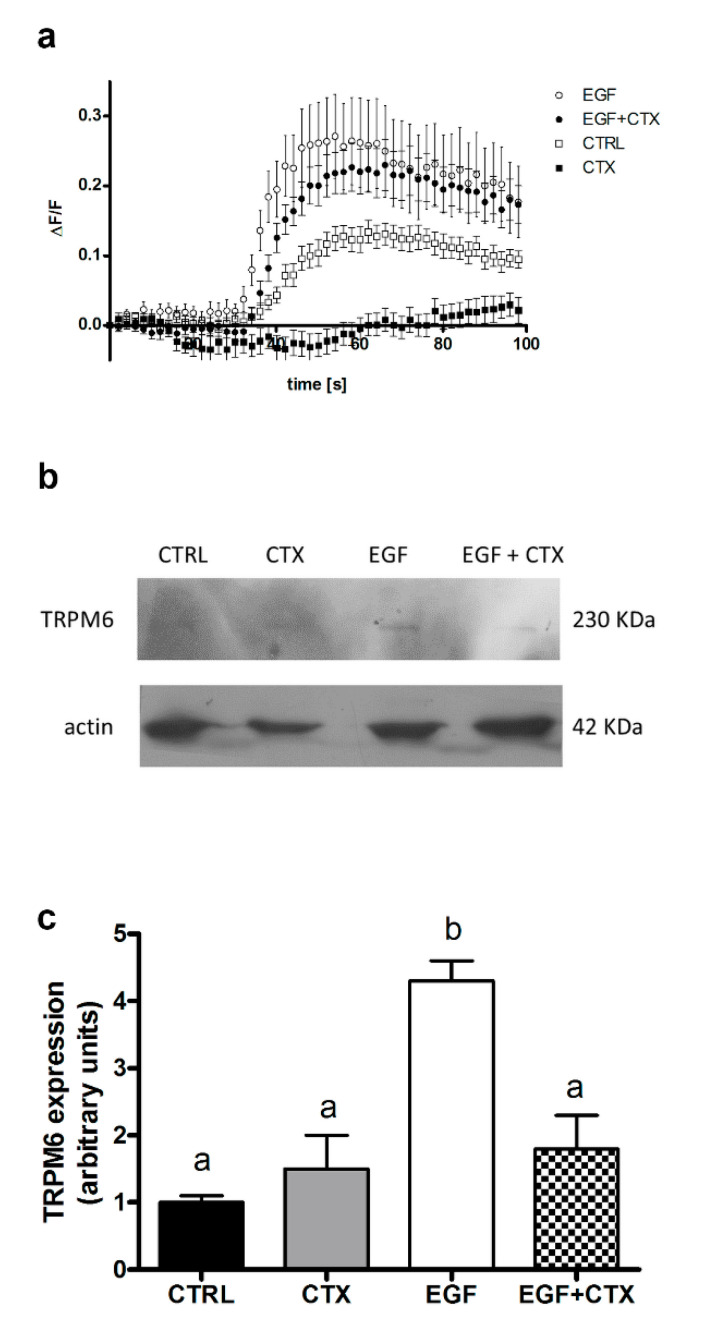
Epidermal growth factor (EGF) stimulates TRPM6 channel expression and Mg influx in human colon carcinoma cells. CaCo-2 cells were serum-starved for 24 h and exposed to EGF (10 ng/mL) and cetuximab (CTX, 70 μg/mL), either alone or in combination, for a further 24h. (**a**) Mg influx capacity, as assessed by the live imaging of Mag-Fluo-4-loaded cells; a representative experiment is shown. (**b**) TRPM6 protein expression, as evaluated by Western blot analysis; a representative blot is shown. (**c**) Quantification of TRPM6 protein expression by Western blot densitometry normalized to β-actin levels (*n* = 3, mean ± SD). Data sharing the same letter are not significantly different (*p* > 0.05) according to one-way ANOVA, followed by Bonferroni’s test. Full scans of original blots are available in Figure S2.

Molecular crosstalk between the EFGR pathway and TRPM6 has been described in kidney epithelial cells [21,22]. We sought to assess whether the same mechanisms may modulate the Mg influx in intestinal epithelial cells. As shown in Figure 2a, EGF stimulation (10 ng/mL, 24 h) induced an increase in the basal Mg influx capacity of CaCo-2 cells (open circles vs. open squares), while CTX reduced the EGF-dependent increase in Mg influx (solid circles vs. open circles) and completely abrogated the basal Mg influx (solid squares vs. open squares). Western blot analysis proved that 24h of treatment with EGF (10 ng/mL) upregulated the TRPM6 expression, while concomitant exposure to CTX (70 μg/mL) resulted in TRPM6 levels comparable to the basal expression (Figure 2b,c). We conclude that EGF signaling leads to increased levels of the TRPM6 channel in intestinal cells, and CTX, by interfering with this signaling, downregulates the TRPM6-mediated Mg influx.

### 3.3. Mg Supplementation Does Not Affect Cetuximab Efficacy

**Figure 3:**
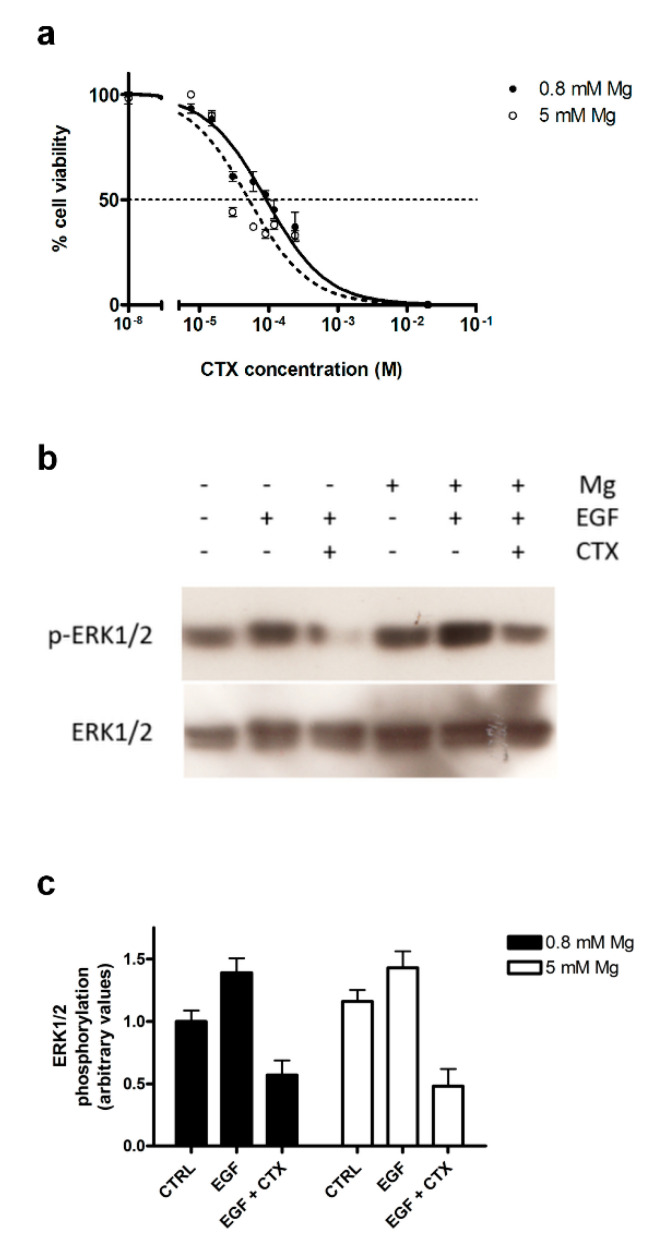
Mg supplementation does not affect cancer cell sensitivity to CTX. (**a**) A representative dose-response curve of CaCo-2 cells exposed to CTX (24 h) in control medium (0.8 mM of MgSO_4_, solid line and circles) or Mg-supplemented medium (5 mM of MgSO_4_, dotted line and open circles). (**b**) Phosphorylation of extracellular signal-regulated kinase (ERK) 1/2 in CaCo2 cells challenged for 24h with EGF (10 ng/mL) with or without CTX (70 μg/mL) in control (0.8 mM of MgSO_4_) or Mg-supplemented (5 mM of MgSO_4_) medium, as assessed by Western blot analysis; a representative blot is shown. (**c**) Quantification of ERK1/2 phosphorylation by Western blot densitometry, normalized to total ERK1/2 levels (*n* = 3, mean ± SD). Two-way ANOVA indicated a very significant effect (*p* < 0.001) of EGF/CTX treatment, while Mg concentration had no significant effect (*p* = 0.70). The effect of CTX on unstimulated cells in control or Mg-supplemented medium is reported in Figure S3.

To evaluate whether the extracellular Mg availability may alter sensitivity to the growth inhibitory effects of CTX, we challenged CaCo-2 cells with CTX in Mg-supplemented (5 mM of MgSO_4_) medium. As shown in Figure 3a, the IC50 for CTX at 24h did not change significantly in the Mg-supplemented cells in comparison to the control cells (50 ± 10 vs. 66 ± 16 μg/mL, respectively). CTX prevents the dimerization of the EGFR and the activation of downstream pathways; primary or acquired resistance to CTX is mainly due to the constitutive activation of MEK signaling with subsequent MAPK activation [30]. Therefore, we also assessed whether Mg supplementation could interfere with the CTX-dependent inhibition of ERK1/2 phosphorylation. Western blot analysis showed that the presence of 5 mM of MgSO_4_ did not substantially change the amount of phospho-ERK1/2 in CTX-treated cells (Figure 3b); two-way ANOVA confirmed that the Mg supplementation had no effect (*p* = 0.70), while CTX treatment had a very significant effect (*p* < 0.001, Figure 3c) on the ERK1/2 phosphorylation.

## 4. Discussion

Cancer-associated hypomagnesemia has long been recognized, and was originally attributed to the metabolic demands of tumor growth; now, it has become clear that also cancer therapies play an important role [13,14]. In contrast to cisplatin, which damages renal tubules and hence causes a generalized electrolyte wasting [3], CTX induces hypomagnesemia by a specific antagonistic effect on renal Mg reabsorption. However, the interference between the mode of action of CTX and the homeostatic mechanisms of Mg has not received the deserved attention to its clinical implications. In this paper, we provide evidence supporting two relevant issues: (1) CTX-induced hypomagnesemia is not just due to renal wasting, but also to impaired intestinal absorption; (2) Mg supplementation does not modify CTX cytotoxicity.

Until recently, renal Mg reabsorption was thought to play a pivotal role in maintaining systemic Mg homeostasis [16]. However, conditional knockout murine models have proved that wild-type kidneys are not able to compensate for the ablation of intestinal TRPM6 and have pointed to an indispensable function of gut Mg absorption in the maintenance of proper Mg status [17]. The data we present here are in line with this view, confirming that cross-talk between EGF and TRPM6 occurs also in intestinal cells. In our work, we focus on the pathological setting of colon cancer and the mechanisms of CTX-induced hypomagnesemia. We are aware that our results do not conclusively prove that the activation of the EGFR pathway regulates the TRPM6-mediated intestinal Mg absorption in a more physiological context; future studies in more appropriate models will address this issue. On the other hand, it could be speculated that, in cancer cells, autocrine signaling by EGF may potentiate the TRPM6-mediated magnesium influx or compete with CTX. However, the EGF production by CaCo-2 cells does not undermine the value of our results, since in our model CTX does inhibit cellular growth as well as both basal and EGF-stimulated magnesium influx.

We propose that the impaired Mg absorption in the intestine heavily contributes to the severe hypomagnesemia that occurs in many CTX-treated patients. In the kidneys, the EGF-dependent modulation of TRPM6 has been ascribed to two different mechanisms: (1) the altered endomembrane trafficking of TRPM6, which results in increased TRPM6 channel activity on the cell surface [22], and (2) a transcriptional effect on the *TRPM6* mRNA expression via the MAPK/ERK pathway [31]. In our colon cellular model, we confirm that EGF increases the TRPM6 protein expression, while we have no evidence that acute EGF stimulation affects the number of recycling channels in favor of increased plasma membrane expression. We are aware of the limitations of our approach resulting from analyzing the TRPM6 protein expression by Western blot. However, the mRNA expression levels might not necessarily translate into a functional channel protein. Indeed, post-translational modifications may affect the protein levels, regardless of (or in addition to) transcriptional events; for example, this has been demonstrated for the sister channel TRPM7 [32]. On the other hand, ion channels are notoriously challenging to study for two main reasons: (a) the paucity of channel molecules per cell—a rough estimate obtained by electrophysiological studies is about 70 molecules/cell (Prof. Andrea Fleig, personal communication); (b) the difficulty of producing specific antibodies, due to structural constraints and sequence similarity. The commercial anti-TRPM6 antibody that we use is the only known antibody that does not display cross-reactivity with TRPM7 [19]. Ultimately, we are interested in the functional effects of cross-talk between EGF and TRPM6; in this respect, by using a functional assay we provide definitive proof that, in intestinal cells, (a) TRPM6 is indispensable for mediating the Mg influx (Figure 1a), and (b) CTX modulates the TRPM6-mediated Mg influx (Figure 2a). 

The symptoms of hypomagnesemia range from depression and muscle spasms to arrhythmias and seizures, and significantly worsen the quality of life of patients [16]. Severe hypomagnesemia warrants treatment by intravenous and/or oral magnesium supplementation for the duration of CTX therapy, and cases of CTX dose reduction and discontinuation have been documented [33]. No evidence-based guidelines have currently been developed for the management of hypomagnesaemia in the context of CTX cancer therapy [33]. We demonstrate that CTX inhibits the TRPM6-mediated transcellular Mg transport. However, oral Mg supplementation, by increasing the intraluminal Mg concentration and thus favoring the paracellular route, may represent an effective strategy to restore the Mg status in CTX-treated patients, even in the presence of limited renal transcellular reabsorption. Most importantly, such approach would be feasible on an outpatient basis. 

Although most medical oncologists agree on the necessity of restoring the Mg status in symptomatic patients [31], the relationship between Mg and cancer remains highly controversial [34]. Early reports found that, in murine models, a low Mg availability resulted in the inhibition of primary tumor growth, but at the same time enhanced metastasis formation [35,36]. On the other hand, more recently Mg status was reported to have no influence on tumor progression in two different animal models [37,38]. Notably, Mg supplementation protected against cisplatin-induced acute kidney injury without compromising the cisplatin-mediated killing of an ovarian tumor xenograft in mice [37]. Despite the limitations of an in vitro model, our data oppose the view that the effect of Mg deficiency on cell proliferation is synergistic with that of CTX: Mg supplementation did not significantly affect cell growth (Figure S4), nor did it alter the effect of CTX inhibitory activity on cell growth or on MAPK signaling (Figure 3).

In conclusion, we present evidence that Mg supplementation does not compromise CTX efficacy, and suggest that nutritional intervention may be a safe and cost-effective approach by which to maximize patient wellbeing and improve CRC management. Further preclinical and clinical research will be necessary to clarify the relationship between Mg and CTX in various experimental tumor models and the potential of Mg supplementation in CTX-treated patients.

## Supplementary Materials

The following are available online at https://www.mdpi.com/2072-6643/12/11/3277/s1: Figure S1: full scan of the original blot for TRPM6 shown in Figure 1; Figure S2: full scan of the original blot for TRPM6 shown in Figure 2; Figure S3: phosphorylation of ERK1/2 in unstimulated CaCo2 treated with CTX in control or Mg-supplemented medium; Figure S4: growth curve of CaCo-2 cells in control or Mg-supplemented medium.
