# Supplementary material for: Magnesium Absorption in Intestinal Cells: Evidence of Cross-Talk between EGF and TRPM6 and Novel Implications for Cetuximab Therapy
Source: Nutrients. 2020 Oct 26;12(11):3277. doi: 10.3390/nu12113277 (PMC7692710; doi:10.3390/nu12113277)
Supplement: Supplementary file 1 [file nutrients-12-03277-s001.pdf]

Supplementary Materials to "Magnesium Absorption in Intestinal Cells: Evidence of Cross-Talk between EGF and TRPM6 and Novel Implications for Cetuximab Therapy" by Pietropaolo et al.

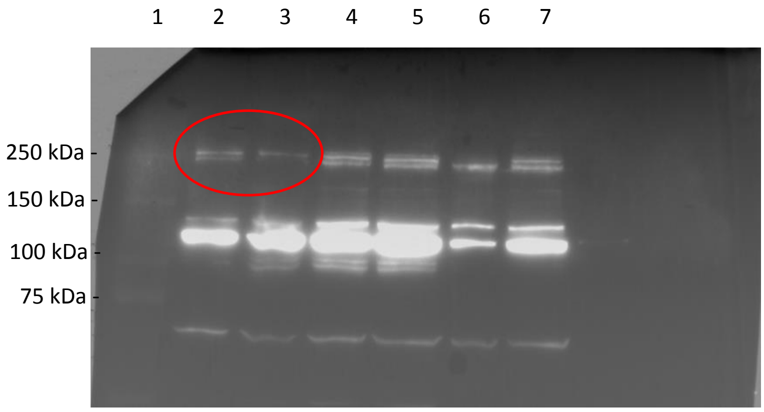

Figure S1. Full scan of the original blot for TRPM6 shown in Figure 1. In lane 1, molecular weight markers are faintly visible. Lanes from 4 to 7 contain irrelevant samples. Lanes 2 and 3 contain control and *TRPM6*-silenced CaCo-2 cells, respectively. Commercial anti-TRPM6 antibodies recognize several unspecific bands, most notably the ones between 100 and 150 kDa. However, according to the expected molecular weight for TRPM6 (around 230 kDa) and our previous results [19], we analyzed the uppermost band.

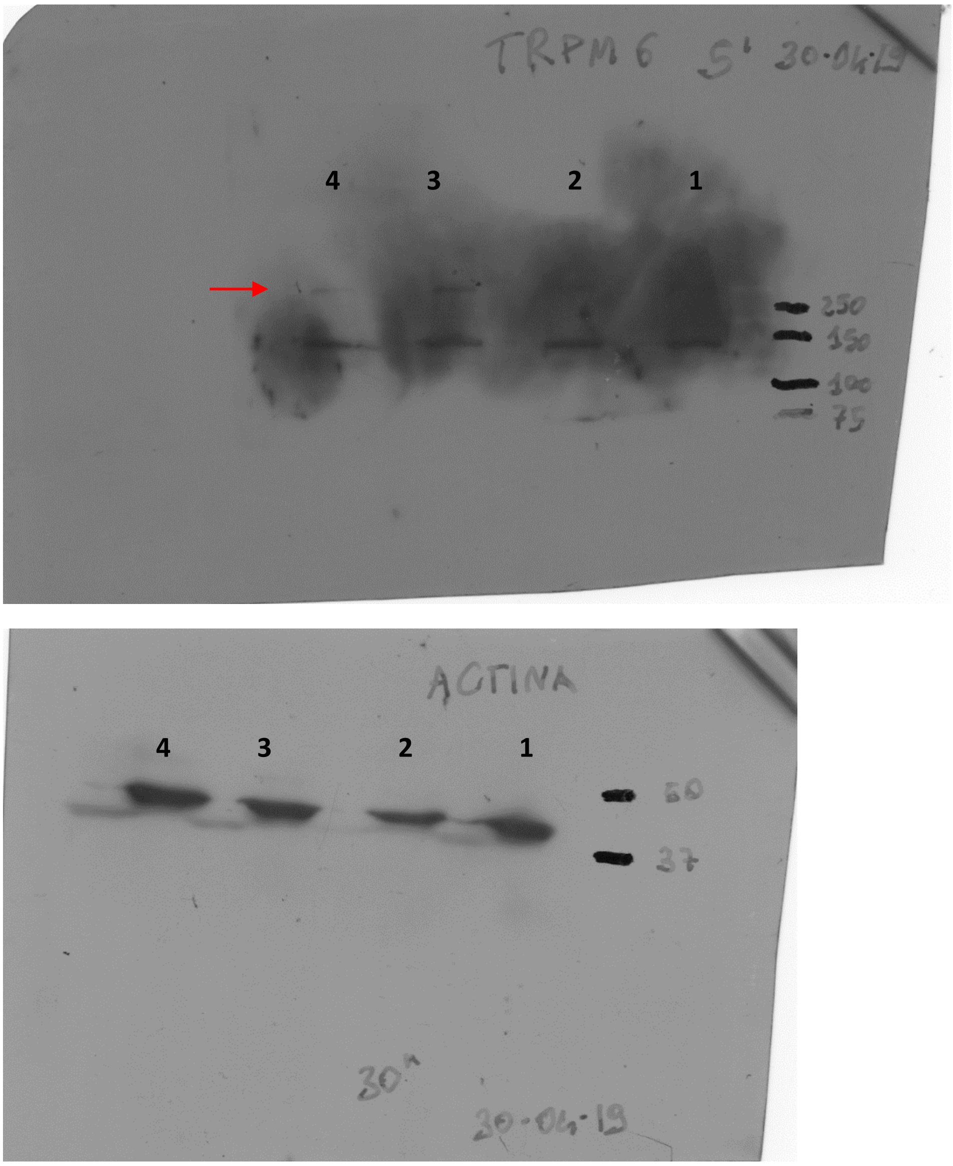

Figure S2. Full scan of the original blots for TRPM6 (above) and actin (below) shown in Figure 2. 1: Control CaCo-2 cells; 2: CTX-treated CaCo-2 cells; 3: EGF-treated CaCo-2 cells; 4: CaCo-2 cells treated with both EGF and CTX. Commercial anti-TRPM6 antibodies recognize several unspecific bands, most notably the ones between 100 and 150 kDa. However, according to the expected molecular weight for TRPM6 (around 230 kDa) and our previous results [19], we analyzed the uppermost band.

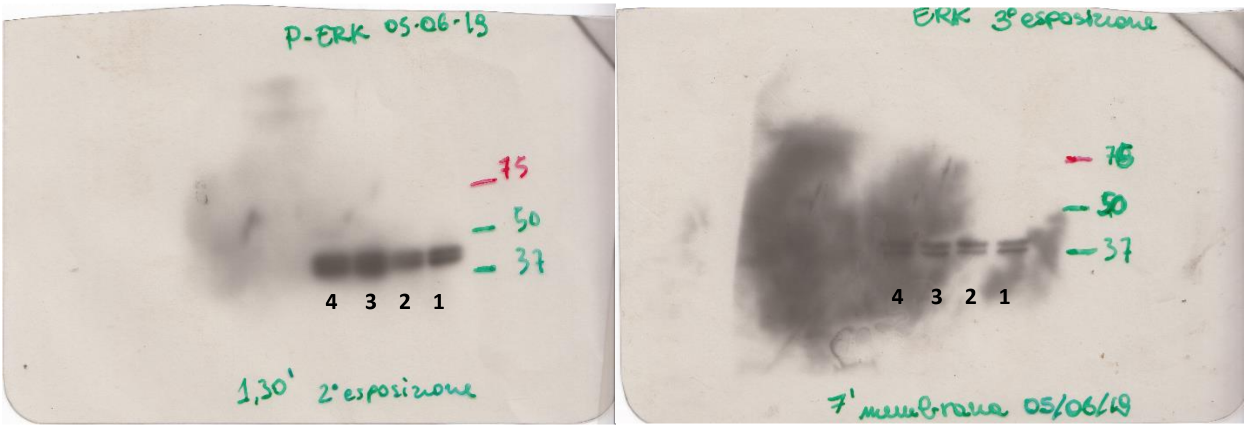

Figure S3. Phosphorylation of ERK1/2 in unstimulated CaCo-2 cells treated with CTX in control or Mg-supplemented medium. Full scans of the original blots are shown. 1: Control cells in 0.8 mM Mg medium; 2: CTX-treated cells in 0.8 mM Mg medium; 3: Control cells in 5 mM Mg medium; 4: CTX-treated cells in 5 mM Mg medium. The effect of CTX is less evident because cells are serum starved and were not exposed to EGF.

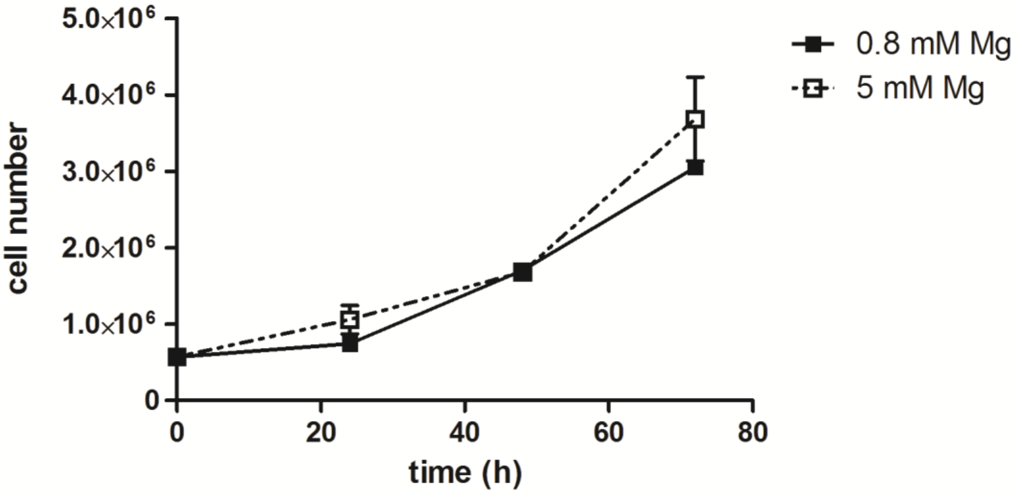

Figure S4. Growth curve of CaCo-2 cells in control (0.8 mM $MgSO_4$) or Mg-supplemented (5 mM $MgSO_4$) medium.
